# Leptin favors imbalance of antigen-specific CD4^+^ T-cells associated with severity of cat allergy

**DOI:** 10.3389/fimmu.2023.1290740

**Published:** 2023-10-26

**Authors:** Carolina Vollmer, Aleida Dias, Marisa Sales, Priscila M. Sacramento, Júlio Cesar Silva, Hugo A. A. Oyamada, Ulisses C. Linhares, Sudhir Gupta, Taissa M. Kasahara, Cleonice A. M. Bento

**Affiliations:** ^1^ Post-graduate Program in Cellular and Molecular Biology, Federal University of the State of Rio de Janeiro, Rio de Janeiro, Brazil; ^2^ Department of Microbiology and Parasitology, Federal University of the State of Rio de Janeiro, Rio de Janeiro, Brazil; ^3^ Post-graduate Program in Microbiology, University of the State of Rio de Janeiro, Rio de Janeiro, Brazil; ^4^ Department of Morphological Sciences, Federal University of the State of Rio de Janeiro, Rio de Janeiro, Brazil; ^5^ Department of Medicine, University of California, Irvine, Irvine, CA, United States

**Keywords:** leptin, Fel d1, Th2/Th9, Tfh cells, Treg/Tr-1 cells, Tfr cells

## Abstract

**Introduction:**

Obesity can complicate IgE-mediated allergic diseases. In the present study, we aimed to investigate the ability of obesity-related concentrations of leptin to modulate the *in vitro* effector and regulatory Fel d1-specific CD4^+^ T-cell subsets in patients allergic to cat, considered the third most common cause of respiratory allergy in humans.

**Methods:**

For this study, plasma and peripheral blood mononuclear cells (PBMC) from 30 cat-allergic patients with mild, moderate and severe respiratory symptoms were obtained. The PBMC cultures were stimulated with Fel d1 antigen (10 µg/mL) in the presence or absence of obesity-related leptin dose (50 ηg/mL). After 6 days, the levels of cytokines and IgE in the supernatants were evaluated by multiplex and ELISA, respectively. The frequency of different non-follicular (CXCR5^-^) and follicular (CXCR5^+^) Fel d1-specific CD4^+^ T cell subsets was determined by flow cytometry. The plasma levels of leptin and IgE anti-cat titers were evaluated by ELISA and ImmunoCAP, respectively.

**Results and conclusions:**

Fel d1 induced both IgE production and release of cytokines related to Th2, Th9 and Th17 cell phenotypes. Feld1 was more efficient in increasing the frequency of T_FH_IL-21^-^ cells positive for IL-4, IL-5 and IL-13 than T_FH_IL-21^+^ cell subsets. Leptin favored the expansion Th2-like and Th9-like cells and T_FH_IL-21^-^ cells positive for IL-4, IL-5 and IL-13, but reduced the proportion of conventional (Treg/Tr-1) and follicular (T_FR_) regulatory CD4^+^ T-cell subsets expressing or not CD39 marker. Finally, many of the imbalances between Fel d1-specific CD4^+^ T-cells were also correlated with plasma leptin and anti-Fel d1 IgE titers. In summary, hyperleptinemia should negatively impact on the severity of cat allergies by favoring the expansion of pathogenic Fel d1-specific CD4^+^ T-cell phenotypes and damaging the functional status of regulatory CD4^+^ T-cell subsets.

## 1 Introduction

Cat allergies are the most common mammalian‐origin allergy in humans, affecting approximately 1 in 5 adults worldwide (1, 2). The most common clinical presentations in these patients are rhinitis, asthma, and/or conjunctivitis. When persistent, the clinical symptoms may impair quality of life (3, 4). Furthermore, severely allergic patients may present an anaphylactic reaction, requiring emergency medical care. Although eight allergens derived from cats have been described, designated Fel d1 to d8, only Fel d1 has clinical significance, accounting for up to 96% of allergic sensitization to cats in humans (5, 6). Primarily produced by salivary and sebaceous glands (5). Fel d1 can easily become and remain airborne in dander and dust particles for extended periods (5, 6).

The hallmark of cat sensitization and symptom severity is the production of high‐affinity Fel d1-specific IgE (6, 7). Although Th2 cytokines, IL-4 and IL-13, can increase IgE production, the synthesis of this antibody is critically dependent on B cell collaboration with follicular helper T (T_FH_) cells (8). T_FH_ cells are specialized CD4^+^ T cells that provide help to B cells activation into germinal center (GC) of lymphoid follicles. In the GC, T_FH_ cells are characterized by high expression of CXCR5, programmed cell death protein (PD-1), B cell lymphoma 6 (Bcl-6), and IL-21 production (9). The main function of CXCR5 is to guide T_FH_ cells migration towards lymphoid follicles in response to its ligand, the CXCL13, abundantly produced by GC-derived B cells (9). On the other hand, IL-21 from T_FH_ cells not only mediate the selection of high**-**affinity and isotype switched B cells, but also promote differentiation of these lymphocytes into plasma cells and memory B cells (9). Although T_FH_ cells in peripheral blood are Bcl-6 negative and express low PD-1 levels, they are able to induce antibody production from peripheral B cells (9). Based on cytokines, human circulating T_FH_ cells have been classified as T_FH_1 (IL-21^+^IFN-γ^+^), T_FH_2 (IL-21^+^IL-4^+^), T_FH_17 (IL-21^+^IL-17^+^) and, more recently, T_FH_13 (IL-21^low^L-4^hi^IL-5^hi^IL-13^hi^) (8).

Many studies have demonstrated the involvement of T_FH_2 cells in the pathogenesis of allergic IgE-mediated airway diseases (10–16). In patients suffering from allergic rhinitis and asthma, elevated frequency of circulating T_FH_2 has been associated with plasma IgE titers and clinical exacerbation (10–14). Moreover, the expansion of T_FH_2 cells inside the airways of allergic patients appear to promote IgE production by local activated B cells, which may play an important role in mast cells and eosinophil activation (15, 16).

Interestingly, more recent studies have demonstrated that, while T_FH_2 cells induce low-affinity IgE production, the synthesis of high-affinity IgE to allergens critically depends on T_FH_13 cells (8, 17). The binding of high-affinity IgE/allergen to FcεRI on mast cells and basophils immediately triggers histamine release, quickly causing a cluster of typical cat allergic symptoms (18). Further, in addition to mast cells, eosinophils activated by the same Fel d1/IgE complexes contribute to allergy pathogenesis by producing lager amounts of leukotrienes (LTC4, LTD4, and LTE4) and platelet-activating factor (PAF), pro-inflammatory lipids that induce local vasodilatation, edema, neurogenic stimulation, smooth muscle contraction and hypersecretion of mucus (19). Moreover, IL-9-secreting CD4^+^ T (Th9) cells have also been implicated in atopic allergy (20). IL-9 prolongs the survival of mast cells, potentiates IgE production and amplifies the ability of IL-5 and IL-13 to increase eosinophil survival and mucus production (20).

As well as inducing the Th2/T_FH_2/T_FH_13 axis, the severity of IgE-mediated allergies has been associated with functional impairment of non-follicular [Treg (CXR5^-^FoxP3^+^IL-10^+^) and Tr-1 (CXCR5^-^FoxP3^-^IL-10^+^)] and follicular [T_FR_ (CXCR5^+^ FoxP3^+^IL-10^+^)] regulatory CD4^+^ T cells (21, 22). While T_FR_ cells control IgE production by B cells in GCs, Treg and Tr-1 cells are essential to reducing inflammatory cytokine release by local mast cells, eosinophils and Th2 cells (21, 22). Therefore, any adverse event that favors Th2/T_FH_ cell expansion and damages regulatory CD4^+^ T cell phenotypes should affect the severity of atopic diseases, such as obesity.

Obesity has been related to severity of allergy symptoms and to higher levels of total and allergen-specific IgE in atopic individuals (23, 24). In cat allergic patients, obesity was associated with total and allergen-specific IgE levels (25). This adverse relationship must be, at least partially, associated with high leptin production, an adipokine known to modulate the functional status of T cells (26).

Leptin is a 16 kDa peptide encoded by the OB gene. At physiological concentrations, leptin plays an adjuvant role in the immune response against different pathogens (27). However, hyperleptinemia, as observed in obese individuals, has been correlated with the severity of allergic reactions (28). Ciprandi et al. (29) demonstrated a direct relationship between IgE titers and eosinophil counts with leptin levels in patients with allergic rhinitis. With regard to CD4^+^ T cell phenotypes, studies published by our group performed in patients with allergic asthma (AA) have found a positive correlation between plasma leptin levels and circulating Th2- and Th17-like cells able to produce high levels of IL-5, IL-6 and IL-17 in response to mitogen (30, 31). In addition, the frequency of these pro-inflammatory CD4^+^ T cell subsets were directly associated with lung function impairment (31). Still according to our previous study, in CD4^+^ T cell cultures from lean AA patients, obesity-related leptin concentration enhanced Th2- and Th17-related cytokine production and impaired Treg function in response to polyclonal activators (31). However, studies regarding the effects of leptin on the composition of different allergen-specific CD4^+^ T-cells have not been conducted to date. Therefore, the main objective of the present study was to investigate the ability of obesity-related leptin doses to modulate the *in vitro* different effector and regulatory Fel d1-specific CD4^+^ T cells from patients with persistent cat allergies.

## 2 Materials and methods

### 2.1 Subjects

Thirty patients with allergic rhinitis (AR) and/or asthma (AA) to cat dander were recruited from March 2020 to September 2021 from the Federal University of the State of Rio de Janeiro Hospital/UNIRIO (Rio de Janeiro, Brazil). All patients had a skin-prick test and IgE positive for cat dander extract (**Table 1**). Since AA is a disorder characterized by inflammation of the airways and recurrent episode of breathing difficulties triggered by allergens, among our patients, persistent AA was diagnosed by a history of recurrent wheezing, dyspnea and chest tightness, and confirmed by methacholine bronchial hyperresponsiveness, when FEV1 was ≥ 70%, or bronchial reversibility after salbutamol inhalation (when FEV1 was <70%). According to daily frequency, severity of clinical exacerbation, lung function damage and need to hospital admission, AA is classified as mild, moderate or severe (21). Also, interference in daily activities is also taken into account (21). With regard to AR, symptom severity was determined by using the total nasal symptom score (TNSS), which is calculated as the sum of scores for each of nasal congestion, sneezing, nasal itching, and rhinorrhea at each time point, using a four point scale (0–3), where 0 indicates no symptoms, 1 for mild symptoms, 2 for awareness of symptoms (but tolerable), and score 3 for severe symptoms that are hard to tolerate and interfere with daily activity (32) (**Table 1**). We excluded patients taking oral or intravenous steroids, theophylline, long-acting β2-agonists, leukotriene antagonists or antihistamines 1 month prior to the study. As control group, twenty healthy subjects (HS), matched for age and sex and with no history of allergic diseases, were also recruited into the study. According to the body mass index (BMI), subjects were stratified as lean (BMI from 18.5 to 24.9), overweight (BMI from 25 to 29.9) and class I obesity (BMI from 30 to 35). Regardless of experimental group, smoking individuals and those with history of upper or lower airway infectious disease 2 months prior to recruitment were also excluded of the study. The Ethics Committee for Research on Human Subjects at the Federal University of the State of Rio de Janeiro (CAAE 44951215.6.0000.5258), approved the study, and blood was collected only after written informed consent was obtained from each individual.

**Table 1:** The characteristics of subjects.

	Control^1^	CAP^2^		
		*Rhinitis*	*Asthma*	*Asthma and rhinitis*
N^0^ of subjects (n)	20	8	6	16
Gender (female/male) (n)	15/5	6/2	4/2	10/6
Age [(years), mean ± SD]	29.1 ± 13.8	28.8 ± 7.9	31.2 ± 10.1	30.3 ± 8.7
Clinical presentation (n)^3^				
Mild	ND	1	2	1
Moderate	ND	4	3	7
Severe	ND	3	1	8
BMI (n)^4^				
*Lean*	5	2	2	3
*Overweight*	10	3	3	6
*Obese class I*	5	3	1	7

^1^Healthy subjects. ^2^Cat allergic patients suffering from rhinitis, asthma alone or rhinitis and asthma to cat dander. ^3^The severity of rhinitis and asthma symptom was determined by TNSS (total nasal symptom score) and GINA (Global Initiative for Asthma) criteria, respectively. ^4^Body mass index: a value derived from the mass (weight in Kg) and height (in meters) of an individual (lean: 18.5-24.9, overweight: 25-29.9 and obese class I: 30-35). ND, no determined.

### 2.2 Cell cultures

Peripheral blood was collected in heparin-containing tubes (BD Vacutainer, Franklin Lakes, NY) and peripheral blood mononuclear cells (PBMC) were obtained by centrifugation on the Ficoll–Hypaque density gradient. Fresh viable PBMCs (1 × 10^6^/mL) were cultured in 24-well flat-bottomed microplates with 2 mL of RPMI medium (ThermoFisher Scientific Inc.) supplemented with 2 μM of L-glutamine (GIBCO, Carlsbad, CA, USA), 10% fetal calf serum, 20 U/mL of penicillin, 20 μg/mL of streptomycin and 20 mM of HEPES buffer. As positive control, PBMC cultures were stimulated with phytohaemaglutinine (PHA, 1 µg/mL) (Sigma-Aldrich Co) for 3 days in a humidified 5% CO_2_ incubator. In order to evaluate the antigen-specific response, the cells were stimulated with Fel d1 (10 µg/mL) (MyBioSource, San Diego, CA, USA) for 6 days. This concentration of Fel d1 was chosen from a previous study that evaluated T cell response to this antigen (6). In these cultures, the role of leptin (Sigma-Aldrich Co) was determined after the addition of 50 ng/mL of this adipokine. This leptin concentration was determined after a dose-response curve (10, 50 and 100 ng/mL) of cytokine-secreting CD4^+^ T cells from healthy subjects (HS) and cat-allergic patients (CAP) in PBMC cultures activated with PHA (1 µg/mL) (Sigma-Aldrich Co) (**Figure S1**). Notably, this leptin concentration is related to the levels of this adipokine in obese subjects (33). After 6 days of culturing, the supernatants were collected, frozen at -20°C for further analysis of cytokine production (Luminex) and IgE levels (ELISA). The PBMC was also used to identify different CD4^+^ T cell phenotypes using flow cytometry.

### 2.3 Flow cytometry analysis

Different CD4^+^ T cell subsets in response to Fel d1 were identified by staining the PBMCs with mouse anti-human monoclonal antibodies (mAbs) for CD3-APC-H7 (SK7 clone), CD4-BV605 (T4 clone), CXCR5-PerCP.eF710 (mu5ubee clone), PD-1-APC (MIH4 clone), CD39-FITC (TU66 clone), FoxP3-PECy5.5 (PGH101 clone), IL-4-PECy7 (8D48 clone), IL-5-eFluor450 (TRFK5 clone), IL-9-BV4211 (MH9A3 clone), IL-10-BV722 (JES3-9D7 clone), IL-13-APC (JES10-5A2 clone), IL-17-AF488 (eBio64DEC17 clone) and IL-21-PE (3A3-N2.1 clone). These mAbs and all isotype control antibodies were purchased from Thermo Fischer (San Diego, CA, USA). Briefly, PBMCs were incubated with various combinations of mAbs for surface markers (CD3, CD4, CXCR5, PD-1, and CD39) for 30 min at room temperature in the dark, according to manufacturer’s instructions. The cells were washed with PBS + 2%FBS, then submitted to permeabilization by incubating the PBMCs with Cytofix/Cytoperm solution (BD Pharmigen, San Diego, CA) at 4°C for 20 min. After washing, the mAbs for intracellular staining (FoxP3, IL-4, IL-5, IL-9, IL-10, IL-13, IL-17, and IL-21) were added in different combinations and incubated for 30 min at 4°C. The stained cells were acquired on Attune NxT flow cytometers (Thermo Fisher Corporation) and analyzed using FlowJo (Tree Star, Inc). Isotype control antibodies and single-stained samples were used to periodically check the settings and gates on the flow cytometer. After acquisition of 200,000 to 300,000 events, lymphocytes were gated based on forward and side scatter properties after the exclusion of dead cells, by using propidium iodide, and doublets.

### 2.4 Luminex, ImmunoCAP and ELISA assays

The titers of plasma IgE anti-cat was determined by florescence enzyme immunoassay with capsulated cellular polymer solid-phase (ImmunoCAP) coupled with cat dander (REF 14.451201, Therm Fischer Sicentific Inc.) with the detection limit ranging from 0.1 to 100 Ku/L. The cut-off value for IgE positivity was considered 0.35 Ku/L. Circulating leptin levels were measured using a commercial ELISA kit following manufacturer’s instructions (Enzo Life Sciences, Farmingdale, NY). Plates were read at 450 nm in ELISA reader (Dynex Technologies, USA). Lyophilized leptin ranging from 31.3-2000 pg/mL was used to construct the standard curve. The levels of different cytokines and IgE in the supernatants from cell cultures were determined using the “Th1/Th2/Th9/Th17 Cytokine 18-plex human Panel” kit (InvitroGen, San Diego, CA, USA) and human IgE ELISA kit (88-50610-22) (Invitrogen, Thermo Fisher Scientific Co), respectively.

### 2.5 Statistical analyses

All statistical analyses were conducted using the Prism 8.0 program (GraphPad Software). Comparisons between immune assays in non-stimulated (none) or activated PBMC cultures with Fel d1 and Fel d1/leptin were performed with one-way ANOVA, followed by Tukey test for data with Gaussian distribution, and by Kruskal-Wallis, followed by Dunn’s test for data without Gaussian distribution. The nonparametric Mann-Whitney U test and Student’s t test were applied to determine whether the two groups were statistically different for nonparametric and parametric variables, respectively. Pearson’s and Spearman’s correlation were applied for variables with or without normal distribution, respectively. Significance for all experiments was defined as p<0.05.

## 3 Results

### 3.1 Leptin modulates cytokine and IgE production by Fel d1-stimulated PBMCs from cat-allergic patients

**Figure 1:**
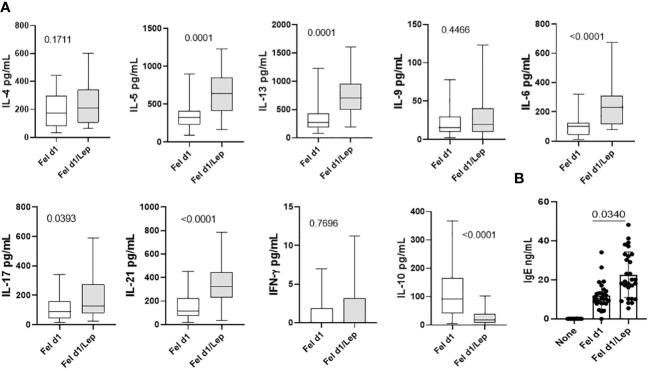
Leptin modulates the cytokine profile and IgE production by PBMCs from cat-allergic patients in response to Fel 1d. PBMC cultures (1 x 10^6^/mL) from cat-allergic patients (n=30) were maintained for 6 days in the presence of culture medium alone (without) or with 10 μg/mL of Fel 1d, with or without 50 ng/mL of leptin (Lep). At the end of the culture time, the supernatants were harvested and the **(A)** cytokine (IL-4, IL-5, IL-13, IL-6, IL-17, IL-21, IFN-γ and IL-10) and **(B)** IgE levels were determined by Luminex and ELISA, respectively. Mean values ​​were compared using one-way ANOVA and *p* values ​​are shown in the graphs. All data are shown as mean ± SD of six independent experiments with 4 and 6 samples per experiment.

**Table 1** shows that most cat allergic patients were overweight/obese women who presented moderate or severe symptoms of rhinitis (AR) and asthma (AA). As no statistical difference among patients with different clinical symptoms (AR x AA x AR/AA) was observed with regard to immunological assays, they were all included together as a single patient group (CAP- cat allergic patients). For the control group, some experiments were additionally performed in 20 age- and gender-matched healthy subjects (HS). Higher levels of IL-4, IL-5, IL-6, IL-13 and IL-17 were observed in CAP-derived PBMC cultures containing plolyclonlly-activated T cells, as compared with HS (**Figure S2**). In those cell cultures, leptin elevated the release of IL-6, IFN-γ and IL-17 in HS group and the secretion of IL-5, IL-6, IL-13 and IL-17 in CAP group. In contrast, this adipokine reduced the levels of IL-10 secreted by mitogen-activated T cells from both experimental groups (**Figure S2**). Concerning the cytokine profile in response to Fel d1, this major cat antigen induced not only the production of IL-4, IL-5, IL-13, IL-9, IL-6, IL-17, IL-21 and IL-10 (**Figure 1A**), but also the secretion of IgE (**Figure 1B**). The addition of leptin increased the release of IL-5, IL-13, IL-6, IL-17, IL-21 (**Figure 1A**) and IgE (**Figure 1B**), but reduced IL-10 production (**Figure 1A**). Of note, neither medium nor leptin alone were not able to induce detectable cytokine (data not shown). Concerning the control group, Fel d1 only significantly elevated the production of IL-10, with no difference after leptin addition (**Figure S3**). In patients, *in vitro* IgE production directly correlated with both IL-4 and IL-5, released by Fel d1-stimulated cells (**Table 2**), and IL-4, IL-5 and IL-9, secreted by Fel d1/Lep-activated PBMC cultures (**Table 2**). In contrast, in Fel d1/Lep-stimulated cells, IL-10 secretion inversely correlated to IgE (**Table 2**).

**Table 2:** Correlation between *in vitro* total IgE production and cytokine profile in Fel d1-stimulated PBMC cultures from cat-allergic patients.

	IgE (ng/mL)			
	Fel d1		Fel d1/Lep	
Cytokines (pg/mL)	*r*	*p (n=30)*	*r*	*p (n=30)*
*IL-4*	0.7324	**0.0002**	0.5343	**0.0152**
*IL-5*	0.7715	**0.0001**	0.4618	**0.0404**
*IL-6*	0.1533	0.5187	0.1026	0.6610
*IL-9*	0.2980	0.2020	0.5648	**0.0095**
*IL-13*	0.4045	0.0769	0.3488	0.1320
*IL-17*	0.3263	0.1603	0.2286	0.3323
*IL-21*	0.1064	0.6553	0.2211	0.3488
*IL-10*	-0.3143	0.1772	-0.4547	**0.0440**
Non-T_FH_ cells (%)				
*IL-4^+^*	0.3177	0.1017	0.2120	0.2451
*IL-5^+^*	0.2809	0.2012	0.3223	0.1013
*IL-9^+^*	0.4001	0.1003	0.3673	0.1765
*IL-13^+^*	0.3181	0.1091	0.1983	0.3412
*IL-17^+^*	0.3019	0.1288	0.2883	0.2651
*IL-21^+^*	0.2711	0.2188	0.1577	0.4018
IL-21^+^T_FH_ cells (%)				
*IL-4^+^*	0.4011	0.1122	0.4283	0.0657
*IL-5^+^*	0.5891	**0.0073**	0.7848	**0.0001**
*IL-9^+^*	0.4571	0.1011	0.4122	0.1617
*IL-13^+^*	0.4109	0.1891	0.2512	0.4041
*IL-17^+^*	0.3491	0.2019	0.2947	0.3239
IL-21^-^T_FH_ cells (%)				
*IL-4^+^*	0.6012	**0.0085**	0.6966	**0.0023**
*IL-5^+^*	0.5901	**0.0091**	0.6818	**0.0031**
*IL-9^+^*	0.4112	0.0981	0.6474	**0.0085**
*IL-13^+^*	0.4191	0.1187	0.6928	**0.0021**
*IL-17^+^*	0.3191	0.2018	0.2729	0.3654
Treg cells (%)				
*IL-10^+^FoxP3^+^ CD39^-^*	- 0.1134	0.6019	- 0.2759	0.3589
*IL-10^+^FoxP3^+^ CD39^+^*	-0.2133	0.3891	-0.1653	0.5870
Tr-1 cells (%)				
*IL-10^+^FoxP3^-^ CD39^-^*	-0.1411	0.6781	-0.1320	0.6653
*IL-10^+^FoxP3^-^ CD39^+^*	-0.3712	0.1891	-0.4097	0.1335
T_FR_ cells (%)				
*IL-10^+^FoxP3^+^ CD39^-^*	- 0.5781	**0.0212**	- 0.7135	**0.0011**
*IL-10 FoxP3^+^ CD39^+^*	-0.6011	**0.0116**	-0.7552	**0.0003**
*IL-10^+^FoxP3^-^ CD39^-^*	-0.3019	0.2711	-0.4009	0.1645
*IL-10^+^FoxP3^-^CD39^+^*	-0.1092	0.6012	-0.2944	0.3259

In PBMC cultures from cat-allergic patients (n=30) stimulated with Fel d1 or Fel d1/Lep, the levels of cytokines, determined via Luminex, and the frequency of different effector and regulatory CD4^+^ T cells subsets, evaluated by cytometry, was correlated with the in vitro total IgE concentration, assayed through ELISA.

Bold values indicate significance (p<0.05).

### 3.2 Leptin alters the frequency of effector and regulatory Ag-specific CD4^+^ T cell subsets in CAP

**Figure 2:**
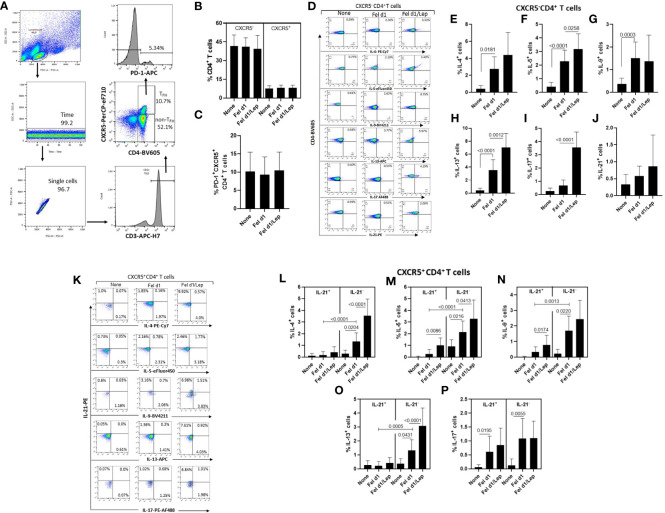
Leptin effect on the frequency of different Fel d1-specific T_FH_ and non-T_FH_ cell subsets in cat-allergic patients. PBMCs (1 x 10^6^/mL) from cat-allergic patients (n=30) were cultured for 6 days in the presence of culture medium alone (none) or with 10 µg/mL Fel 1d, with or without 50 ng/mL leptin (Lep). At the end of the culture time, and adopting the gating strategy shown in graph **(A)**, the mean ± SD of conventional CD4^+^ T cells (non-T_FH_, CXCR5^-^) and total T_FH_ cells (T_FH_, CXCR5+) **(B)**, as well as the T_FH_PD-1^+^ cell subset **(C)** were analyzed by cytometry. In **(D, K)**, representative dot-plots of cytokine-producing non-T_FH_ and T_FH_ cells were shown, respectively. In (**E–J**), the mean ± SD of percentage of **(E)** IL-4^+^, **(F)** IL-5^+^, **(G)** IL-9^+^, **(H)** IL-13^+^, **(I)** IL-17^+^ and **(J)** IL-21^+^ among non-T_FH_ cells, while **(L-P)** showed the mean ± SD values for T_FH_IL-21^+^ and T_FH_IL-21^-^ cells able to produce IL-4 **(L)**, IL-5 **(M)**, IL-9 **(N)**, IL-13 **(O)**, and IL-17 **(P)**. Data are shown as mean ± SD of five independent experiments with 2 and 6 samples per experiment. Significance was calculated using one-way ANOVA and *p* values ​​are shown in the graphs.

From identification of CXCR5 and PD-1 markers on CD4^+^ T cells, and using the gating strategy shown in **Figure 2A**, no difference in the percentage of non-T_FH_ cells (CXCR5^-^) (**Figure 2B**), whole T_FH_ (**Figure 2B**) and T_FH_ PD-1^+^ (**Figure 2C**) cells was observed in the cell cultures stimulated with Fel d1, with or without leptin. In contrast, taking into account the representative dot-plots shown in **Figure 2D**, Fel d1 elevated the proportion of Th2-like cells [IL-4^+^ (**Figure 2E**), IL-5^+^ (**Figure 2F**) and IL-13^+^ (**Figure 2H**)] and Th9 (IL-9^+^) cells (**Figure 2G**), with no change in the percentage of Th17-like cells (IL-17^+^ and IL-21^+^) (**Figures 2I, J**). Leptin elevated the frequency of Fel d1-specific Th2-like cells [IL-5^+^ (**Figure 2F**) and IL-13^+^ (**Figure 2H**)] and Th17-like cells [IL-17^+^ (**Figure 2I**)]. With regards to the classical T_FH_ cells (CXCR5^+^IL-21^+^), and following the gating strategy shown in **Figure 2K**, Fel d1 upregulated the proportion of T_FH_IL-21^+^IL-17^+^ (**Figure 2P**). Notably, Fel d1 more efficiently upregulated the frequency of T_FH_IL-21^-^ cells positive for IL-4 (**Figure 2L**), IL-5 (**Figure 2M**), IL-9 (**Figure 2N**), and IL-13 in comparison with T_FH_IL-21^+^cells (**Figure 2O**). Leptin not only enhanced the proportion of T_FH_IL-21^+^ IL-5^+^ (**Figure 2M**) and T_FH_IL-21^+^ IL-9^+^ (**Figure 2N**), but also that of T_FH_IL-21^-^ cells positive for IL-4 (**Figure 2L**), IL-5 (**Figure 2M**), and IL-13 (**Figure 2O**). The ability of leptin to upregulate non-T_FH_ and T_FH_ cell phenotypes was observed in cell cultures from lean, overweight and obese patients (data not shown).

**Figure 3:**
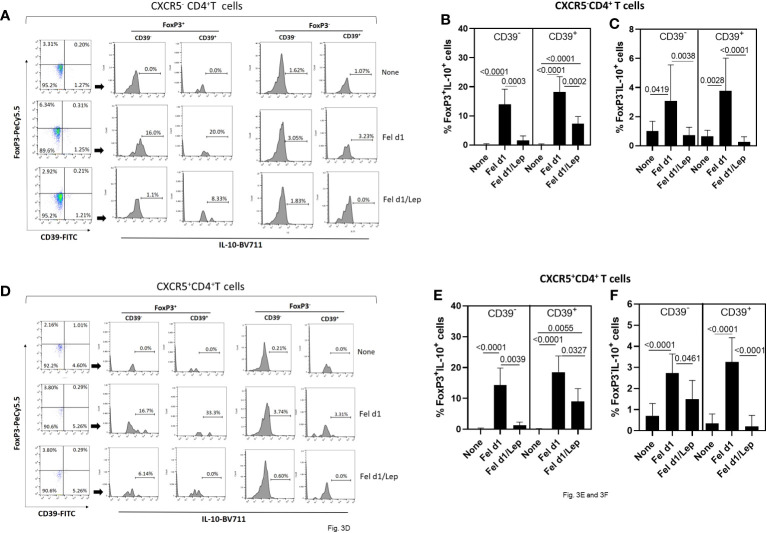
Leptin reduced the frequency of Fel d1-specific Tregs and T_FR_ cell subsets in PBMC cultures from cat-allergic patients. Taking into account the expression of CXCR5, FoxP3, IL-10 and CD39, and following representative dot-plots and histograms shown in graphs **(A, D)**, the frequency of **(A)** conventional (Tregs/Tr-1, CXCR5^-^) and **(D)** follicular (T_FR_/T_FR_-1, CXCR5^+^) regulatory CD4^+^ T cells was determined in PBMC cultures from cat-allergic patients (n=30) after stimulation for 6 days with Fel d1 and Fel d1/Lep. The mean values ( ± SD) of FoxP3^+^IL-10^+^CD39^-^ and FoxP3^+^IL-10^+^CD39^+^ **(B, E)**, and FoxP3^-^IL-10^+^CD39^-^ and FoxP3^-^IL-10^+^CD39^+^ **(C, F)**, on Fel D1-specific Treg and T_FR_ cell subsets was determined. Data are shown as mean ± SD of five independent experiments with 2 and 6 samples per experiment. Significance was calculated using one-way ANOVA and *p* values ​​are shown in the graphs.

Concerning regulatory T cells, through the expression of FoxP3, IL-10 and CD39 on CD4^+^ T cells, we determined the impact of leptin on modulating the proportion of Fel d1-specific Treg/Tr-1 cells (**Figures 3A–C**) and T_FR_ cells (**Figures 3D–F**). Taking into account the gating strategy shown in **Figures 3A, D**, Fel d1 increased the proportion of Treg (CXCR5^-^FoxP3^+^IL-10^+^) (**Figure 3B**) and T_FR_ (CXCR5^+^FoxP3^+^IL-10^+^) (**Figure 3E**), expressing or not CD39. This allergen also upregulated the frequency of Tr-1 (CXCR5^-^FoxP3^-^IL-10^+^CD39^-^ and CXCR5^-^FoxP3^-^IL-10^+^CD39^+^) (**Figure 3C**) and follicular Tr-1-like cells (CXCR5^+^FoxP3^-^IL-10^+^CD39^-^ and CXCR5^+^FoxP3^-^IL-10^+^CD39^+^) (**Figure 3F**). Regardless of cell subtype, leptin significantly reduced the proportion of IL-10-secreting CD4^+^ T cell subsets (**Figure 3**).

*In vitro* IgE production directly correlated with the percentage of T_FH_IL-21^+^IL-5^+^ and T_FH_IL-21^-^ positive for IL-4 and IL-5 in Fel d1- and Fel d1/Lep-stimulated cell cultures (**Table 2**). Similarly, higher IgE production was observed in Fel d1/Lep-activated cell cultures with a higher proportion of T_FH_IL-21^-^IL-9^+^ and IL-21^-^IL-13^+^ (**Table 2**). By contrast, IgE negatively correlated with the proportion of allergen-specific FoxP3^+^IL-10^+^ T_FR_ cells that express, or not, CD39 marker, mainly after leptin addition (**Table 2**). No relationship was observed for the frequency of allergen-specific non-T_FH_ and Treg cells and IgE levels after leptin addition (**Table 2**).

**Figure 4:**
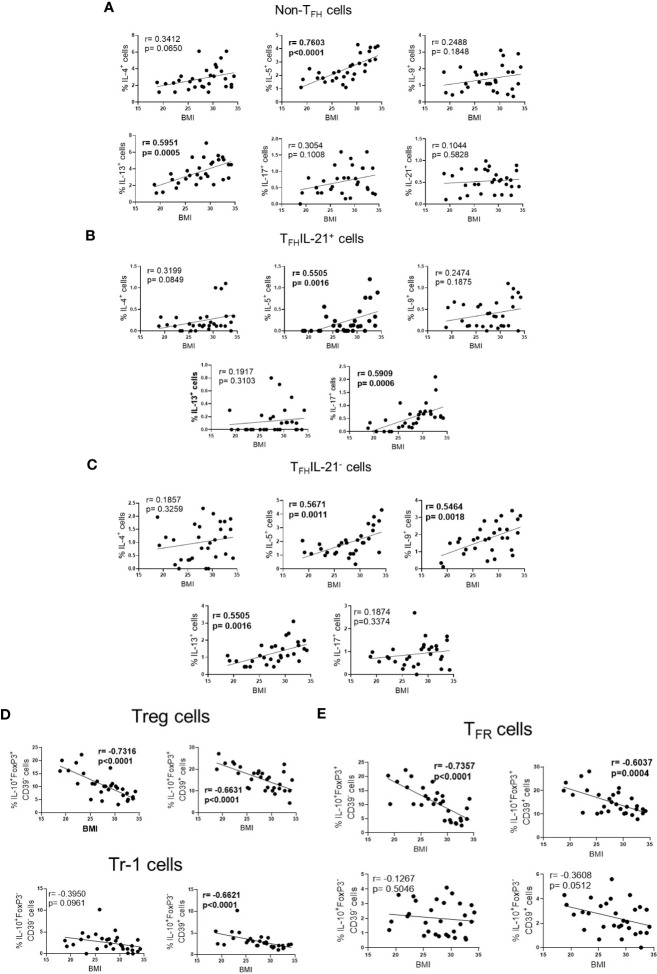
The frequency of effector and regulatory Fel d1-specific CD4^+^ T cell subsets according BMI. In cat-allergic patients (n=30), the frequency of cytokine-producing non-T_FH_ cells **(A)**, IL-21^+^ **(B)** and IL-21^-^ T_FH_ cells **(C)**, as well as Treg/Tr-1 **(D)** and T_FR_ **(E)** cells in response to Fel d1 was correlated with BMI by using Pearson’s correlation.

Finally, according to BMI, higher frequency of Th2-like cells (IL-5^+^ and IL-13^+^) (**Figure 4A**), T_FH_IL-21^+^ (IL-5^+^ and IL-17^+^) (**Figure 4B**) and T_FH_IL-21^-^ cell subsets (IL-5^+^, IL-9^+^ and IL-13^+^) (**Figure 4C**), was observed in obese patients. Conversely, obesity negatively impacted the ability of Fel d1 to elevate Treg (**Figure 4D**) and T_FR_ cells (**Figure 4E**), expressing or not CD39, as well as Tr-1 CD39^+^ cells (**Figure 4D**).

### 3.3 Correlation between plasma leptin levels, anti-cat IgE titers and the *in vitro* cytokine profile in CAP.

As demonstrated in **Table 3**, leptin levels positively correlated with IL-5, IL-6 and IL-17 secretion by Fel d1-stimulated cells, as well as the frequency of both non-T_FH_ (IL-5^+^, IL-13^+^ and IL-17^+^) and T_FH_IL-21^-^ cells positive for IL-5, IL-9 and IL-13. In contrast, a negative correlation was observed between circulating levels of this adipokine and the proportion of Treg and T_FR_ cells, expressing or not CD39 marker. Moreover, the proportion of CD39^+^Tr-1 cells inversely correlated with leptin concentration (**Table 3**). Although no significant correlation was observed between plasma leptin and anti-cat IgE (r=0.4054, p=0.0845), titers of this antibody positively correlated with both IL-5 release and the percentage of T_FH_IL-21^-^IL-4^+^ and IL-21^-^IL-13^+^ cells in Fel d1-stimulated PBMC cultures (**Table 4**). On the other hand, higher levels of this antibody were observed in patients with lower Treg and T_FR_ cell proportions, expressing CD39 or not, and Tr-1 CD39^+^ cells (**Table 4**).

**Table 3:** Correlation between plasma leptin levels and cytokine profile of cells in Fel d1-stimlated PBMC cultures from cat-allergic patients.

	Leptin (ng/mL)	
Cytokines (pg/mL)	*r*	*p (n=30)*
*IL-4*	0.1998	0.3982
*IL-5*	0.6438	**0.0022**
*IL-6*	0.5476	**0.0124**
*IL-9*	0.1674	0.4805
*IL-13*	0.3117	0.1810
*IL-17*	0.4989	**0.0251**
*IL-21*	0.3013	0.1968
*IL-10*	-0.2360	0.3165
Non-T_FH_ cells (%)		
*IL-4^+^*	0.2822	0.3472
*IL-5^+^*	0.7354	**0.0004**
*IL-9^+^*	0.2296	0.4475
*IL-13^+^*	0.6960	**0.0011**
*IL-17^+^*	0.7868	**0.0002**
*IL-21^+^*	0.1816	0.5498
IL-21^+^T_FH_ cells (%)		
*IL-4^+^*	0.3877	0.1897
*IL-5^+^*	0.3052	0.2096
*IL-9^+^*	0.1429	0.6428
*IL-13^+^*	0.1447	0.6341
*IL-17^+^*	0.3714	0.1605
IL-21^-^ T_FH_ cells (%)		
*IL-4^+^*	0.4014	0.0595
*IL-5^+^*	0.6611	**0.0165**
*IL-9^+^*	0.7675	**0.0001**
*IL-13^+^*	0.8748	**<0.0001**
*IL-17^+^*	0.3780	0.1014
Treg cells (%)		
*IL-10^+^FoxP3^+^CD39^-^*	-0.8116	**<0.0012**
*IL-10^+^FoxP3^+^CD39^+^*	-0.7331	**0.0007**
Tr-1 cells (%)		
*IL-10^+^FoxP3^-^CD39^-^*	-04924	0.0893
*IL-10^+^FoxP3^-^CD39^+^*	-0.8186	**<0.0001**
T_FR_ cells (%)		
*FoxP3^+^CD39^-^*	-0.7015	**0.0024**
*FoxP3^+^CD39^+^*	0.7845	**<0.0001**
*FoxP3^-^CD39^-^*	0.3011	0.2659
*FoxP3^-^CD39^+^*	0.4018	0.0950

The levels of cytokines secreted, determined via Luminex, and frequency of different conventional (non-T_FH,_ CXCR5^-^) and follicular helper (T_FH_, CXCR5^+^) CD4^+^ T cell subsets, evaluated by cytometry, in Fel d1-stimulated PBMC cultures from cat-allergic patients (n=30). were correlated with the plasma leptin concentration, assayed through ELISA. By evaluating the expression of CXCR5, FoxP3 and IL-10, we identified the frequency of non-follicular [Treg (CXR5^-^FoxP3^+^IL-10^+^) and Tr-1 (CXCR5^-^FoxP3^-^IL-10^+^)] and follicular [T_FR_ (CXCR5^+^ FoxP3^+^IL-10^+^)] regulatory CD4^+^ T cells.

Bold values indicate significance (p<0.05).

**Table 4:** Correlation between total IgE titers and cytokine profiles of Fel d1-stimlated PBMC cultures from cat-allergic patients.

	*IgE (Ku/L)*	
Cytokines (pg/mL)	*r*	*p (n=30)*
IL-4	*0.2431*	*0.3016*
IL-5	*0.6642*	***0.0014***
IL-6	*0.3848*	*0.0939*
IL-9	*0.1853*	*0.4342*
IL-13	*0.1070*	*0.2160*
IL-17	*0.3757*	*0.1036*
IL-21	*0.3473*	*0.1335*
IL-10	*-0.3304*	*0.1547*
Non-T_FH_ cells (%)		
*IL-4^+^*	0.3928	0.1841
*IL-5^+^*	0.1107	0.7176
*IL-9^+^*	0.3737	0.1736
*IL-13^+^*	0.2311	0.4441
*IL-17^+^*	0.3407	0.2318
*IL-21^+^*	0.3465	0.2671
IL-21^+^T_FH_ cells (%)		
*IL-4^+^*	0.3736	0.2070
*IL-5^+^*	0.4101	0.0987
*IL-9^+^*	0.1484	0.6298
*IL-13^+^*	0.1621	0.5938
*IL-17^+^*	0.3301	0.2686
IL-21^-^T_FH_ cells (%)		
*IL-4^+^*	0.6298	**0.0024**
*IL-5^+^*	0.4170	0.0601
*IL-9^+^*	0.3809	0.1526
*IL-13^+^*	0.6823	**0.0078**
*IL-17^+^*	0.3791	0.2025
Non-T_FH_ cells (%)		
*IL-10^+^FoxP3^+^CD39^-^*	-0.8473	**<0.0001**
*IL-10^+^FoxP3^+^CD39^+^*	- 0.7414	**0.0009**
Tr-1 cells		
*IL-10^+^FoxP3^-^CD39^-^*	- 0.3643	0.1017
*IL-10^+^FoxP3^-^CD39^+^*	- 0.7986	**0.0001**
T_FR_ cells (%)		
*IL-10^+^FoxP3^+^CD39^-^*	-0.7070	**0.0016**
*IL-10^+^FoxP3^+^CD39^+^*	-0.8177	**<0.0000**
*IL-10^+^FoxP3^-^CD39^-^*	0.1648	0.5089
*IL-10^+^FoxP3^-^CD39^+^*	0.1956	0.5189

The titer of plasma total IgE was correlated with both cytokines levels, evaluated by Luminex, and the frequency, determined by cytometry, of different conventional (non-T_FH,_ CXCR5^-^) and follicular helper (T_FH_, CXCR5^+^) CD4^+^ T cell subsets in Fel d1-stimulated PBMC cultures from cat-allergic patients (n=30).

Bold values indicate significance (p<0.05).

## 4 Discussion

Obesity can complicate IgE atopic diseases (23, 34). Here, in cat allergic patients, this adverse relationship should involve, at least in part, increased leptin production that promotes an imbalance between different CD4^+^ T cell phenotypes specific to Fel d1, the major cat allergen.

In the present study, Fel d1 not only increased the production of cytokines related to Th2 and Th9 cells, but also the proportion of antigen-specific T_FH_ cell subsets, mainly IL-21^-^IL-4^+^, IL-21^-^IL-5^+^ and IL-21^-^IL-13^+^. Despite the small sample size in this study, a higher percentage of Fel d1-specific Th2-like cells and T_FH_2/T_FH_13 cell phenotypes was observed among obese patients and directly correlated with plasma leptin levels. *In vitro*, this adipokine directly favored the expansion of Fel d1-specific Th2- and Th9-related phenotypes, as well as elevated the percentage of T_FH_IL-21^+^ (IL-5^+^ and IL-9^+^) and T_FH_IL-21^-^ (IL-4^+^, IL-5^+^ and IL-13^+^) cell subsets. Additionally, leptin elevated IgE production. This finding agrees with a study that demonstrated a direct relationship between leptin levels and IgE production in atopic patients (25). Regarding cell phenotypes, IgE levels in PBMC cultures stimulated with Fel d1/Lep directly correlated with the frequency of T_FH_IL-21^+^IL-5^+^ and T_HF_IL-21^-^ negative for IL-4, IL-5, IL-13 and IL-9, but not Th2-like cells. Furthermore, plasma titers of anti-Fel d1 IgE positively correlated with T_FH_IL-21^-^ positive for IL-5 and IL-13. This finding agrees with studies that demonstrate that T_FH_ cells, but not Th2 cells, are critical for IgE production (8, 17). Among T_FH_ cells, while the T_FH_2 cell subset induces the production of low-affinity IgE (8). the T_FH_13 cell subset is responsible for producing high-affinity IgE (17). T_FH_13 cells are characterized by high IL-4, IL-5 and IL-13 expression associated with very low IL-21 production (25). Yang et al. (35) demonstrated that IL-21 inhibits IgE class-switch recombination in human B cells. Although we did not evaluate either T_FH_ cells that simultaneously express IL-4, IL-5 and IL-13, nor IgE affinity, we believe that the ability of leptin to increase the frequency of Fel d1-specific T_FH_IL- 21^-^ able to produce Th2-related cytokines is one of the mechanisms that this adipokine uses to intensify cat allergy severity. Indeed, the formation of high affinity IgE : FcϵRI complexes on mast cells, by activating Lyn/Syk/LAT-1 axis, promotes intense and immediate histamine release and leukotriene synthesis (18, 35, 36), resulting in associated allergic symptoms, such as airway constriction, increased mucus production, and coughing.

Despite not directly inducing IgE production, Th2 cytokines participate in the pathogenesis of atopic allergic reactions. IL-4 and IL-13 amplify eosinophil and Th2 cell transmigration to the allergen exposure site (37). IL-5 is responsible for increasing eosinophil formation and survival (38). IL-13 increases B cell survival (18) and compromises respiratory function by increasing mucus production in the airways (39). Therefore, leptin ability to increase the frequency of Fel d1-specific Th2-like cells should impact the severity of allergic reactions to cats. Indeed, leptin, by potentiating Th2-mediated response, has been associated with atopic diseases (40). Moreover, here, leptin also favored expansion of Fel d1-specific Th9-like cells. IL-9, along with IL-5 and IL-13, prolong mast cell and eosinophil survival, and increase mucus production (20, 41). Interestingly, despite the lack of data about human T_FH_9 cells, in murine allergy models, these cells support memory IgE^+^ B cell generation (42, 43). Finally, the ability of leptin to upregulate Fel d1-induced IL-6 production may also contribute to IgE synthesis, since IL-6 favors B-cell proliferation, plasma cell survival, and antibody production (44–46).

Recently, the severity of mite-allergic asthma has been associated with Der f3-specific Th17 cells (47). Furthermore, IL-17 directly promoted IgE production by human B cells (48) and favors eosinophil accumulation in mucosa of atopic patients (49). In the present study, although Fel d1 has induced T_FH_IL-17^+^, and leptin amplified this cell subtype, no relationship was observed with either *in vitro* IgE production or plasma anti-Fel d1 IgE. However, it is possible that during disease exacerbation, this cell phenotype may contribute to cat allergy immunopathogenesis by promoting eosinophil infiltration into the airway of patients.

Regarding regulatory CD4^+^ T cells, the severity of allergic reactions has been associated with functional damage of allergen-specific Treg cells (CXCR5^-^FoxP3^+^IL-10^+^), Tr-1 (CXCR5^-^FoxP3^-^IL-10^+^) and, mainly in IgE-mediated disorders, T_FR_ (CXCR5^+^FoxP3^+^IL-10^+^) (21, 22). In atopic patients, severity of the symptoms correlated with both dysfunctional T_FR_ cells and elevated frequency of T_FH_2 cells highly capable of assisting IgE production by allergen-specific B cells (13, 32, 50, 51). Those atopic-derived T_FR_ cells show impaired IL-10 production, a net anti-inflammatory cytokine (32, 50, 52). In addition to IL-10, several surface biomarkers can identify highly functional regulatory T cells, such as CD39 (53). In the present study, leptin reduced the frequency of Fel d1-specific Treg/Tr-1 and T_FR_ cells, most of them expressing CD39. It is known that CD39, along with CD73, metabolizes the extracellular adenosine triphosphate (ATP)/adenosine diphosphate (ADP) into adenosine (ADO), a metabolite which inhibits pro-inflammatory T cell phenotypes (53). A study by Li et al. observed the role of CD39^+^Treg cells in controlling airway inflammation in the murine model of allergic asthma (54). Notably, the frequency of Treg and T_FR_ cells, expressing CD39 or not, inversely correlated with both IgE production in Fel d1/Lep-stimulated cell cultures and plasma anti-Fel d1 IgE titers. In agreement with our findings, a study by Martin-Orozco et al. (55), demonstrated an inverse relationship between FoxP3 expression in the regulatory CD4^+^T cell compartment with serum IgE levels and eosinophilia. Therefore, in the present study, high dose of leptin should negatively impact the prognostic of allergic diseases due to the ability of this adipokine in reducing functional Treg/Tr-1 and T_FR_ cells, CD4 T cell subset implicated in controlling Th2/Th9 and T_FH_2/T_FH_13 axis respectively (21, 22).

## 5 Conclusions

Although preliminary, our findings suggest that hyperleptinemia, by favoring expansion of pathogenic Fel d1-specific CD4^+^ T cells and impairing the functioning of regulatory CD4^+^ T cell subsets, would not only exacerbate disease severity, but also negatively impacts the success of allergen-specific immunotherapies against cat allergies (56, 57).

## Data availability statement

The raw data supporting the conclusions of this article will be made available by the authors, without undue reservation.

## Ethics statement

The studies involving humans were approved by Gaffrée e Guinle university hospital research committee and by the Ethics Committee for Research on Human Subjects at the Federal University of the State of Rio de Janeiro (CAAE 44951215.6.0000.5258). The studies were conducted in accordance with the local legislation and institutional requirements. Blood was collected only after written informed consent was obtained from each individual. The participants provided their written informed consent to participate in this study.

## Author contributions

CV: Investigation, Methodology, Writing – original draft, Writing – review & editing. AD: Investigation, Methodology, Writing – review & editing. MS: Formal Analysis, Investigation, Methodology, Writing – review & editing. PS: Formal Analysis, Investigation, Methodology, Writing – review & editing. JS: Formal Analysis, Investigation, Writing – review & editing. HO: Investigation, Writing – review & editing. UL: Investigation, Writing – review & editing. SG: Conceptualization, Funding acquisition, Investigation, Writing – review & editing. TK: Conceptualization, Supervision, Writing – review & editing. CB: Conceptualization, Funding acquisition, Supervision, Writing – review & editing.
