# Supplementary figures and images for: Leptin favors imbalance of antigen-specific CD4+ T-cells associated with severity of cat allergy
Source: Front Immunol. 2023 Oct 26;14:1290740. doi: 10.3389/fimmu.2023.1290740 (PMC10639137; doi:10.3389/fimmu.2023.1290740)

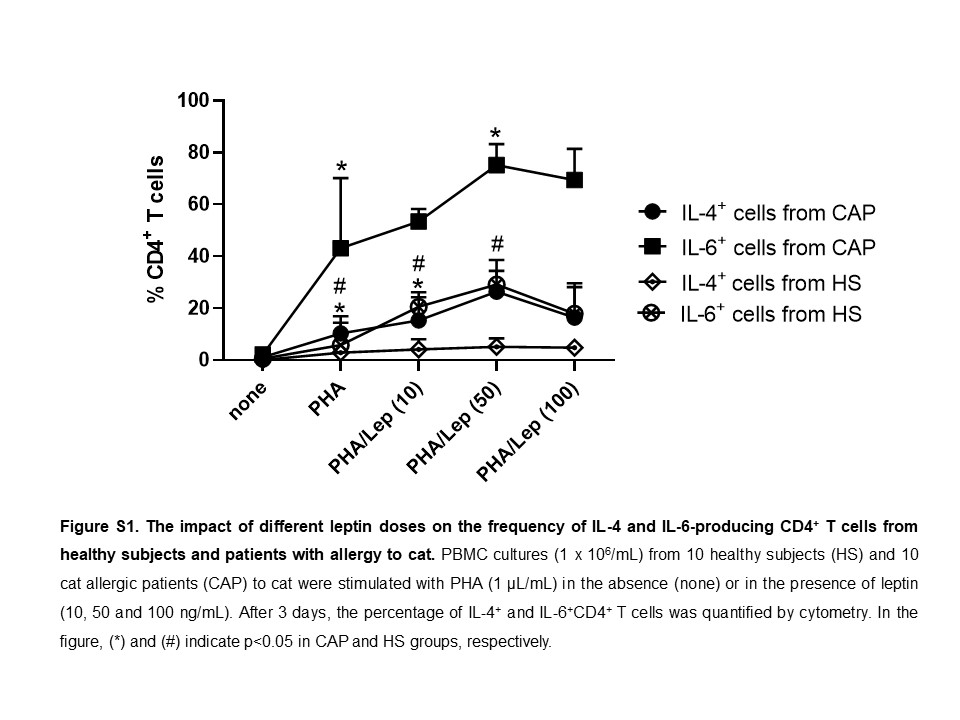

Supplement: Supplementary file 1 [file Image_1.jpeg]

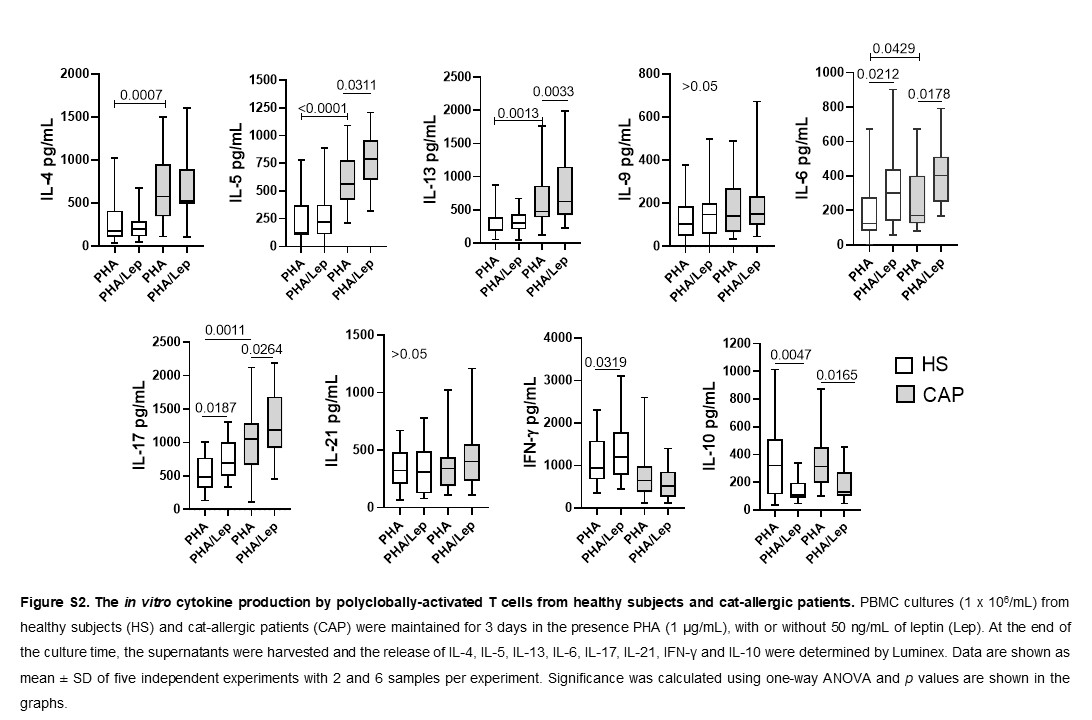

Supplement: Supplementary file 2 [file Image_2.jpeg]

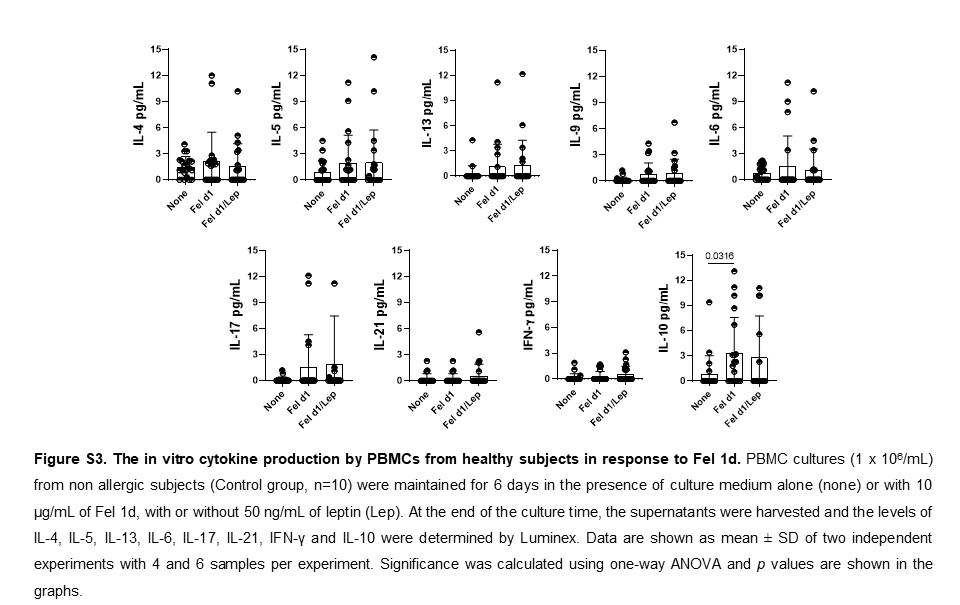

Supplement: Supplementary file 3 [file Image_3.jpeg]
